# Supplementary figures and images for: Contrasting microbial assembly patterns in the woody endosphere of hybrid and non-hybrid Populus trees
Source: PeerJ. 2025 Oct 10;13:e20073. doi: 10.7717/peerj.20073 (PMC12517286; doi:10.7717/peerj.20073)

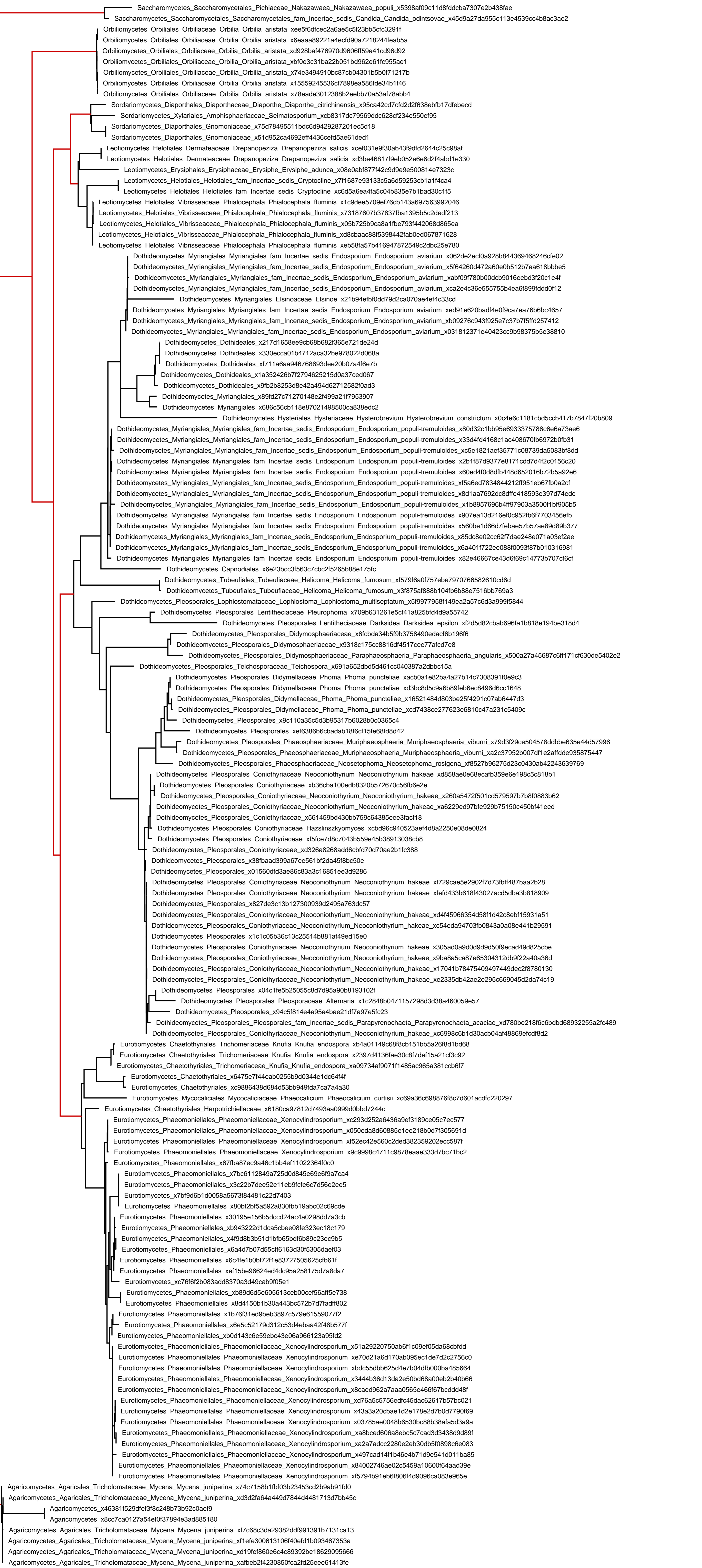

Supplement: Supplemental Information 3 — The tree was generated by grafting ITS1 extension trees (sequences grouped by Class and inferred with IQTREE) onto an ascomycete backbone phylogeny generated by Shen et al. (2018). ITS1 trees were grafted to their corresponding crown node in the backbone phylogeny, with the exception of the basidiomycete tree, which was grafted to the root of the tree. The edges of the backbone tree are highlighted in red. [file peerj-13-20073-s003.pdf]

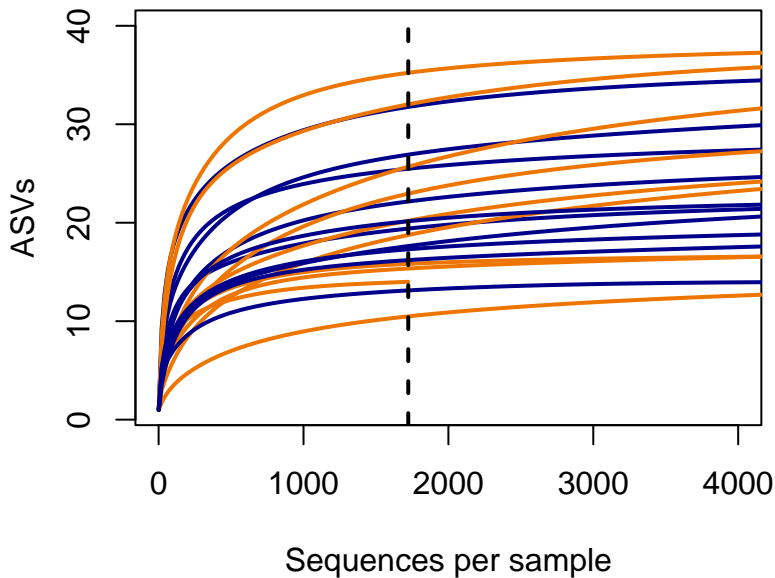

Supplement: Supplemental Information 4 — Colours represent P. deltoides (blue) and P. × jackii (orange) samples. The vertical line indicates the resampling value (i.e., 1,723) selected for fungal community analyses with the ghost tree. [file peerj-13-20073-s004.pdf]

**A.** Host identity: ● *P. deltoides* ● *P. x jackii*

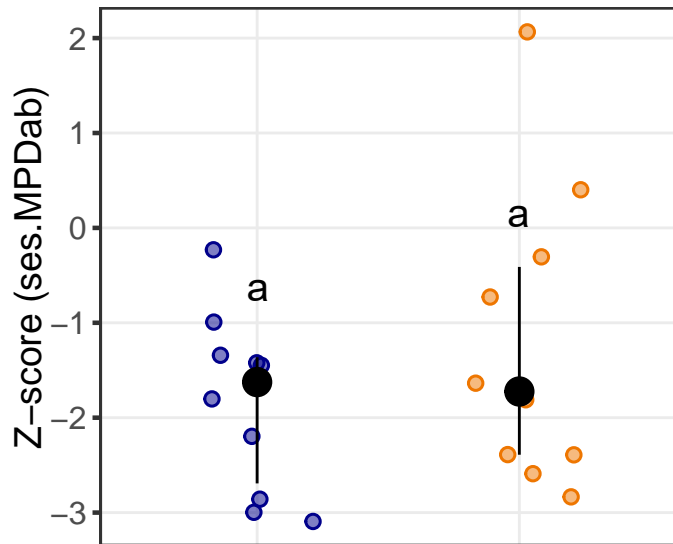

**B.**

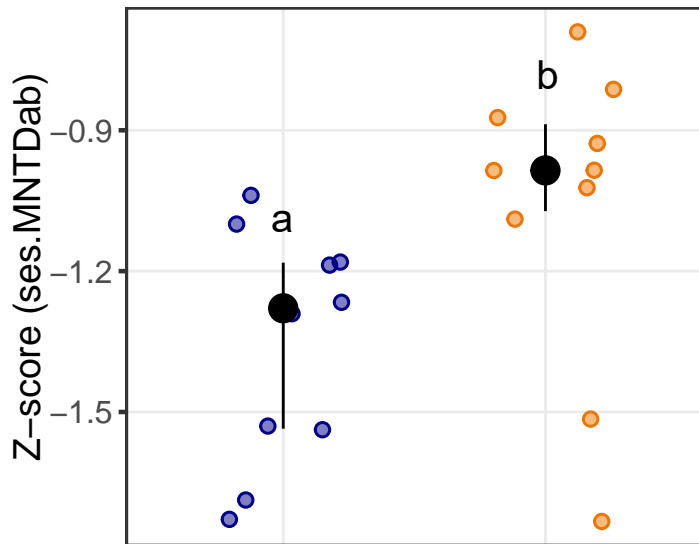

Supplement: Supplemental Information 5 — The phylogenetic dispersion of host-associated fungal communities as measured by ses.MPDab (A) and ses.MNTDab (B) are displayed. Letters within plots indicate significant differences between host taxa (ANOVA at 95% confidence). The interquartile range and median are displayed (black). [file peerj-13-20073-s005.pdf]

**A.**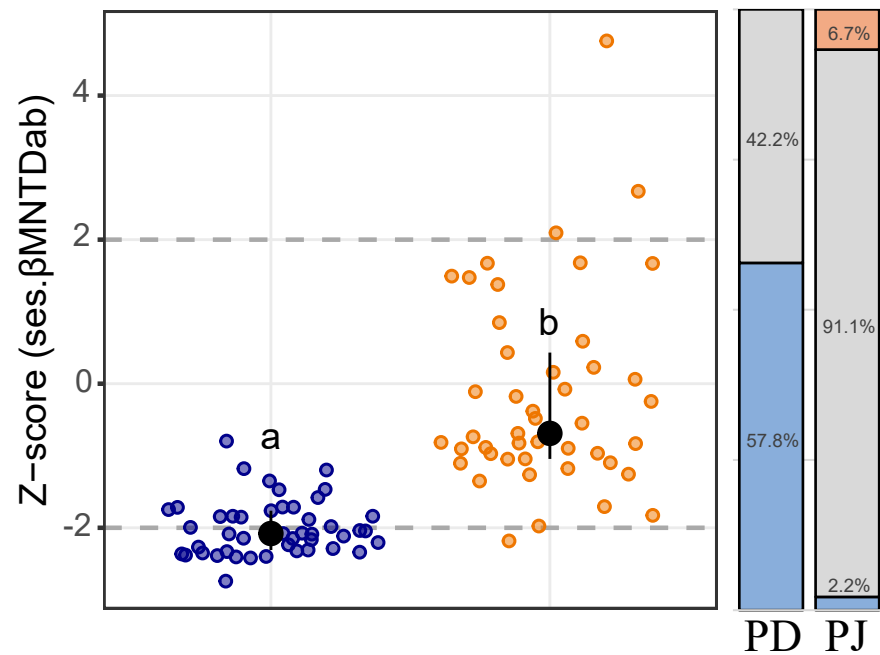**B.**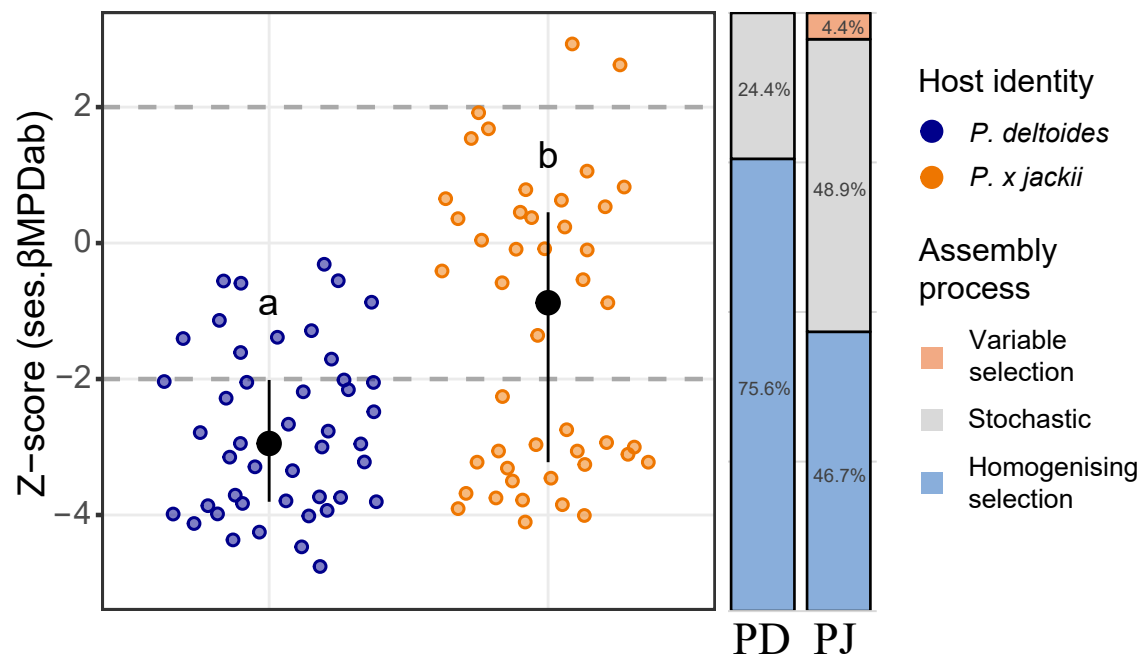

Supplement: Supplemental Information 6 — The phylogenetic beta diversity of host-associated fungal communities, calculated through (A) ses.βMNTDab and (B) ses.βMPDab, are displayed. Points represent pairwise comparisons between samples, expressed as z-scores relative to a null model of random community assembly (see Methods). Letters within plots indicate significant differences between host taxa (ANOVA at 95% confidence), and the interquartile range and median are displayed (black). Grey dashed lines indicate two standard deviations from the mean of the null distribution, beyond which pairwise comparisons are characterised by variable (z-score > +2) or homogenising selection (z-score < −2). Bar charts indicate the percent of pairwise comparisons characterised by different community assembly processes for P. deltoides (PD) and P. × jackii (PJ). [file peerj-13-20073-s006.pdf]

**A.**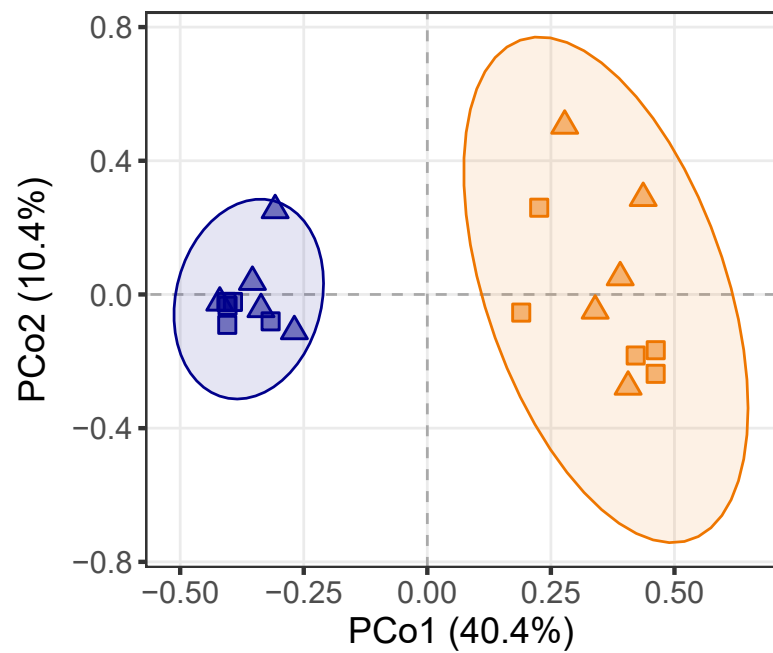**B.**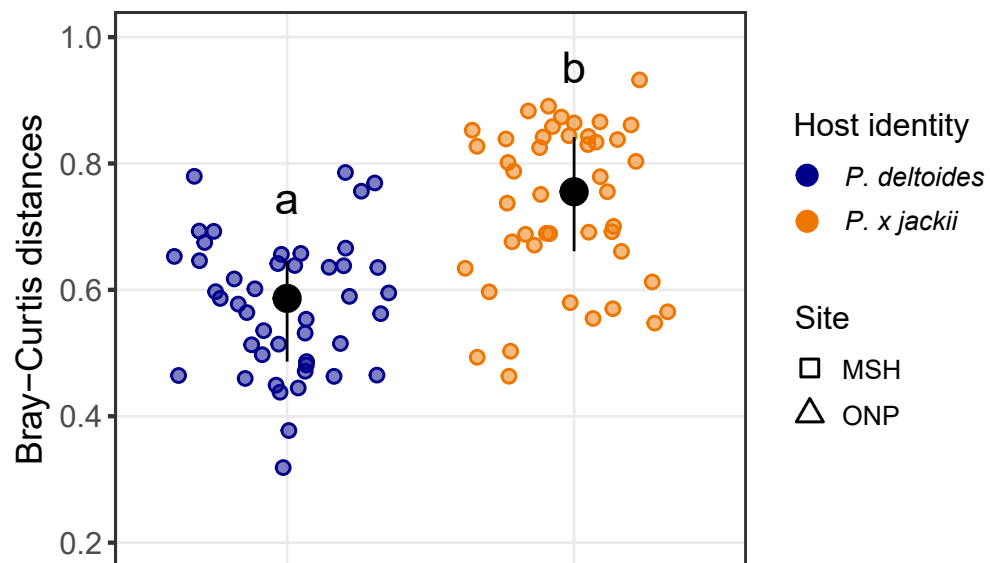**C.**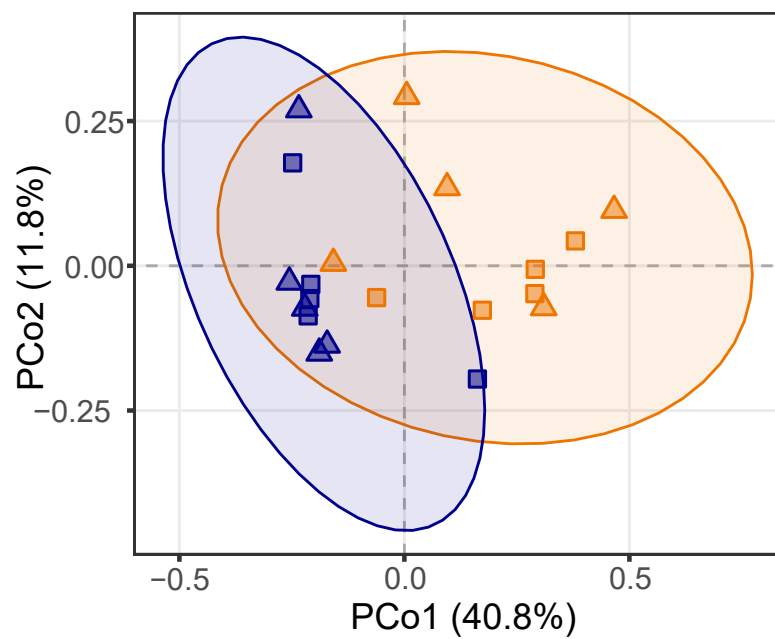**D.**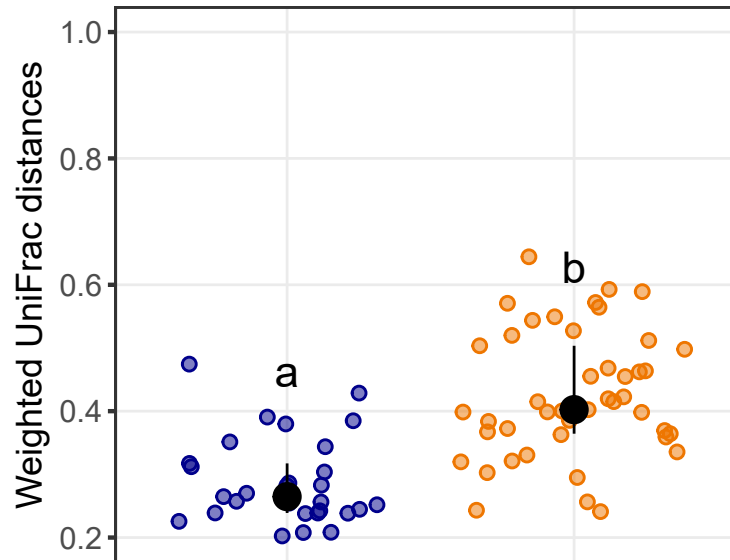

Supplement: Supplemental Information 7 — Principal coordinates analysis (PCoA) of fungal community composition based on (A) Bray–Curtis and (C) weighted UniFrac distances computed with Hellinger transformed data are displayed. (B) Interindividual taxonomic (Bray–Curtis distance) and (D) phylogenetic beta diversity (weighted UniFrac distance) of fungal communities computed with Hellinger transformed data are also displayed. Letters within plots indicate significant differences between host taxa (ANOVA at 95% confidence). The interquartile range and median are displayed (black). [file peerj-13-20073-s007.pdf]

**A.**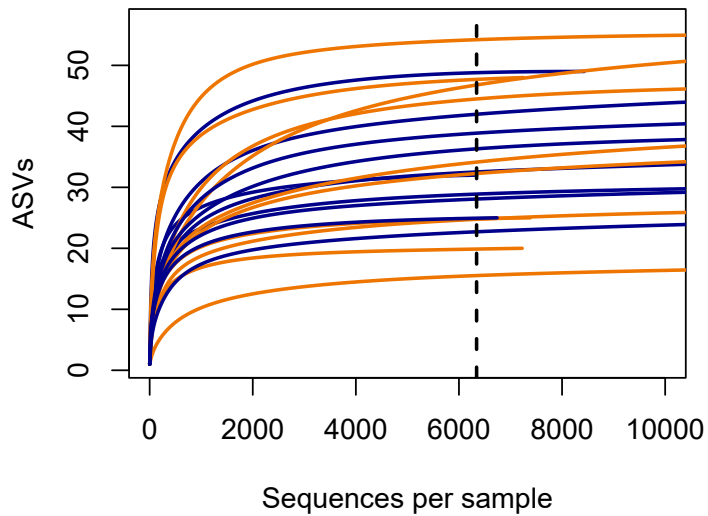**B.**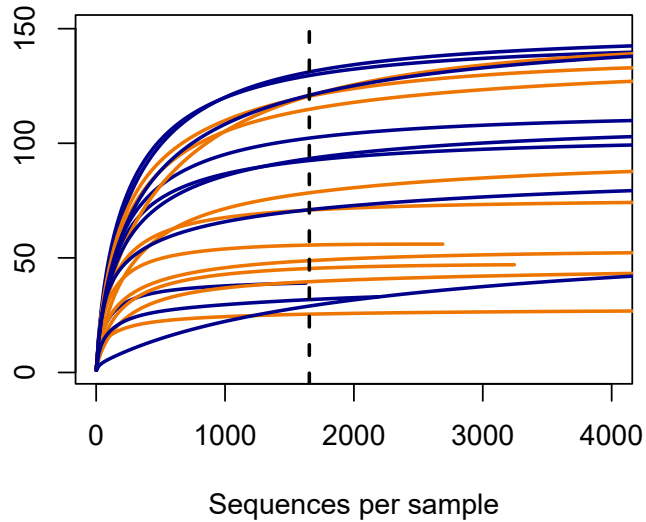

Supplement: Supplemental Information 8 — Rarefaction curves for (A) fungal and (B) bacterial ASVs belonging to P. deltoides and P. × jackii samples are displayed. The vertical line indicates the resampling value selected for fungal (i.e., 6,342) and bacterial (i.e., 1,628) community analyses. [file peerj-13-20073-s008.pdf]

# Bacterial samples

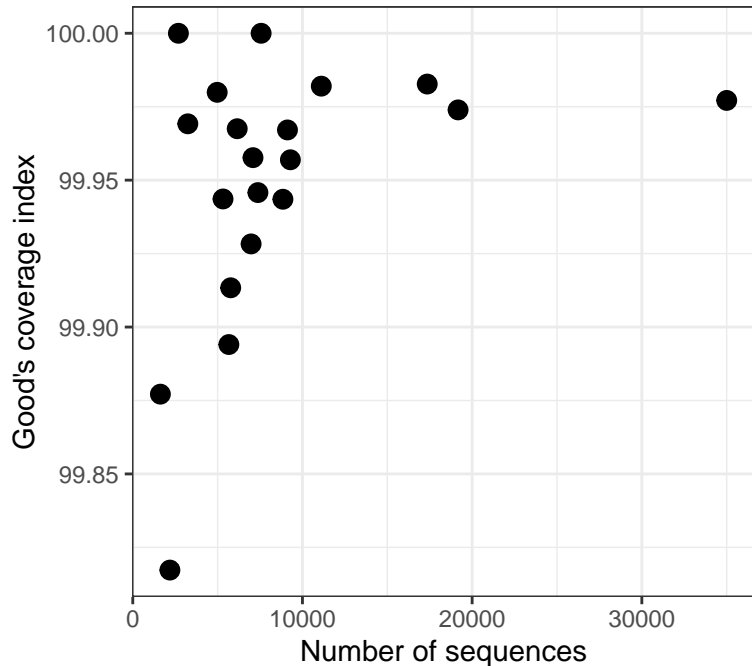

# Fungal samples

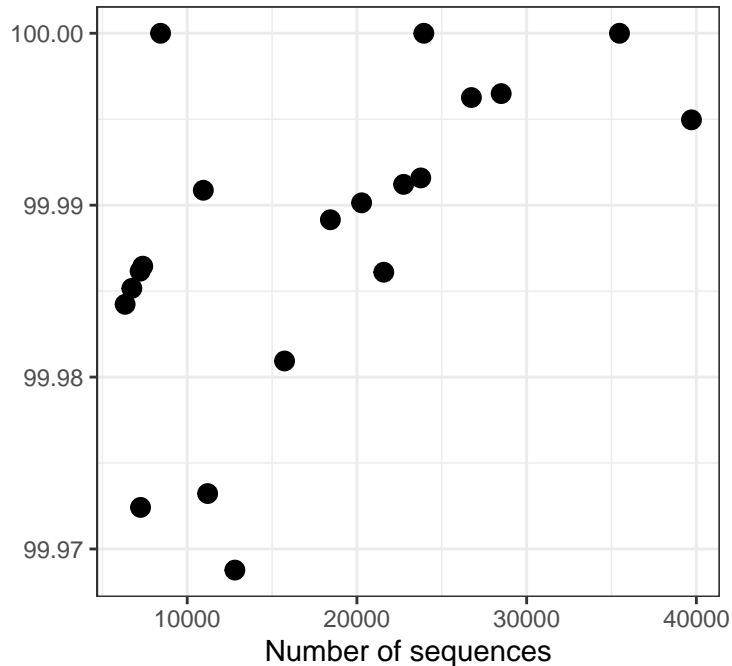

Supplement: Supplemental Information 9 [file peerj-13-20073-s009.pdf]

**A.**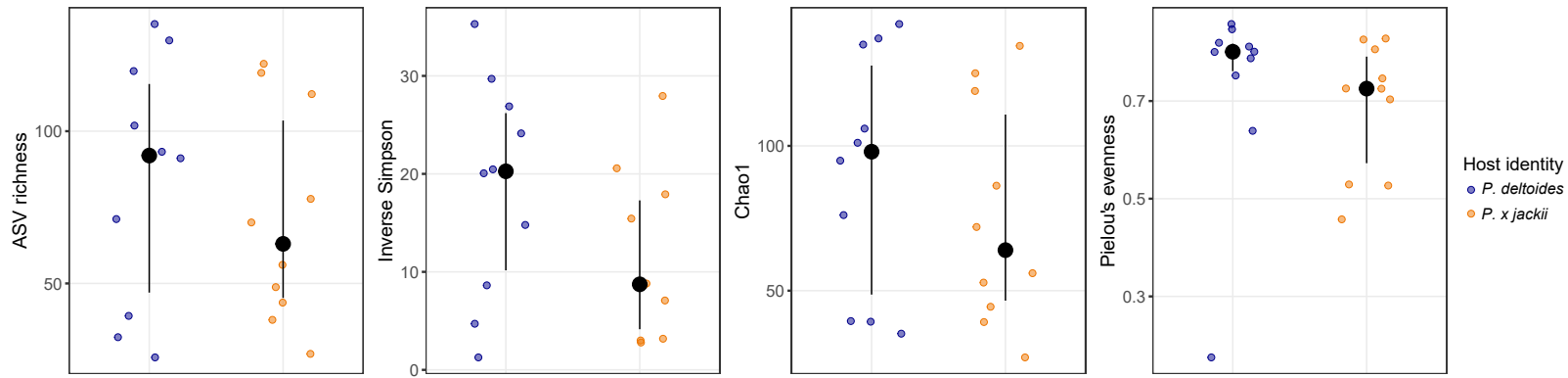**B.**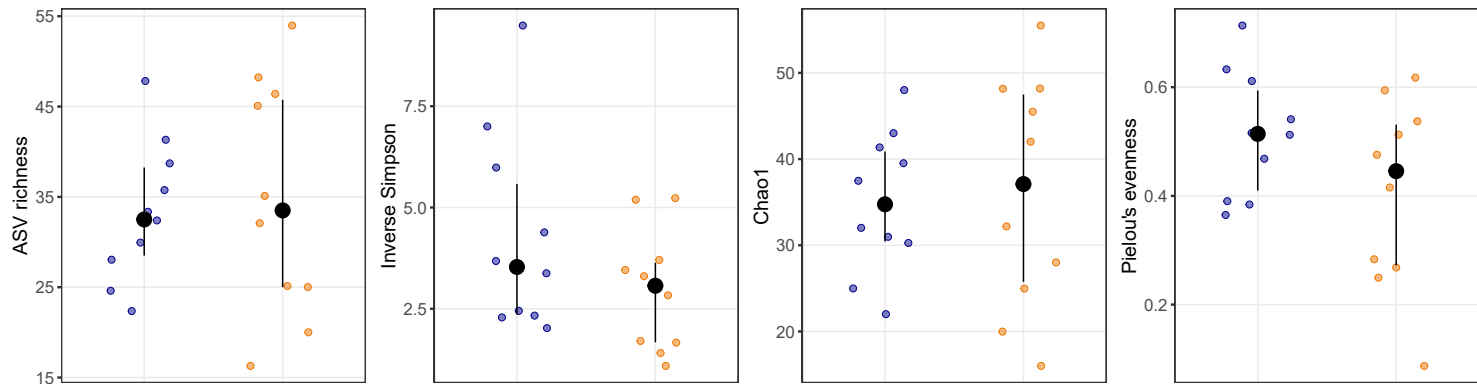

Supplement: Supplemental Information 10 — The taxonomic diversity of host-associated (A) bacterial and (B) fungal communities are displayed. The interquartile range and median are displayed (black). No significant differences were detected between host taxa (ANOVA at 95% confidence). [file peerj-13-20073-s010.pdf]

**A.**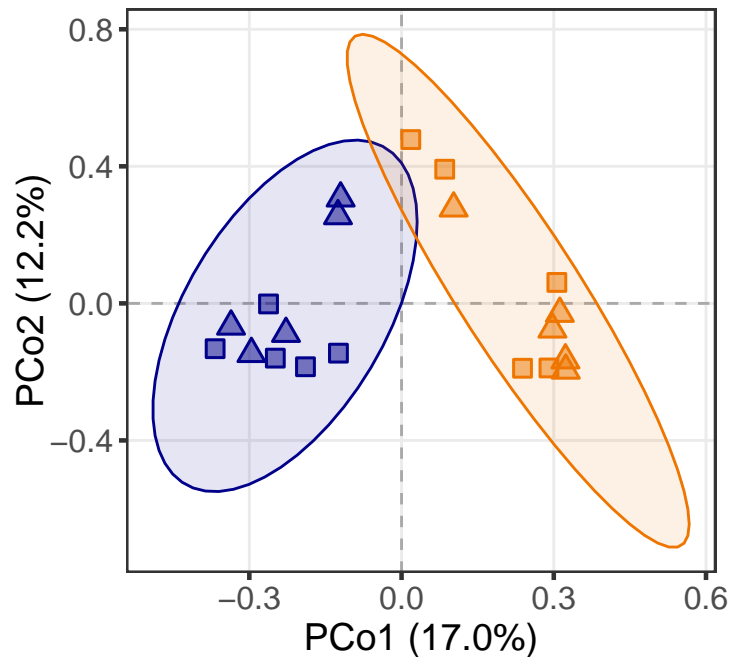**B.**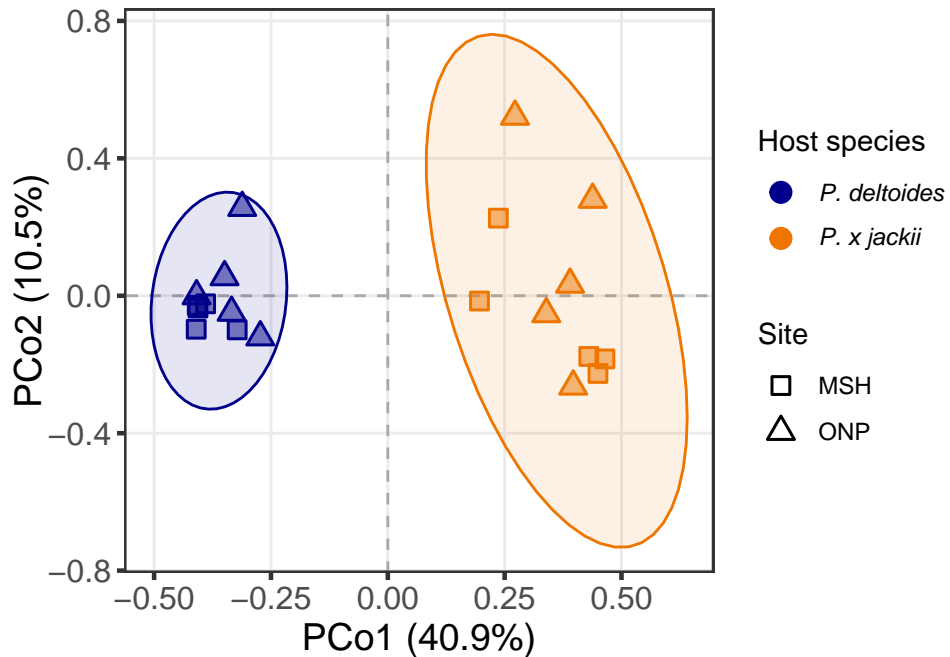

Supplement: Supplemental Information 11 — Principal coordinates analysis (PCoA) of (A) bacterial and (B) fungal community composition based on Bray–Curtis distances computed with Hellinger transformed data are displayed. [file peerj-13-20073-s011.pdf]
