# Supplementary material for: Contrasting microbial assembly patterns in the woody endosphere of hybrid and non-hybrid Populus trees
Source: PeerJ. 2025 Oct 10;13:e20073. doi: 10.7717/peerj.20073 (PMC12517286; doi:10.7717/peerj.20073)
Supplement: Supplemental Information 12 — The community matrices were Hellinger transformed prior to computing Bray–Curtis and weighted UniFrac distances. [file peerj-13-20073-s012.docx]

|  | **Bray-Curtis distance** | | | **Weighted UniFrac distance** | | |
| --- | --- | --- | --- | --- | --- | --- |
| **Model** | **F-value** | **R^2^** | **p-value** | **F-value** | **R^2^** | **p-value** |
| **Fungal community** |  |  |  |  |  |  |
| *Host identity* | **15.092** | **0.440** | **<0.001** | **12.748** | **0.424** | **<0.001** |
| *Sites* | 1.903 | 0.055 | 0.099 | 1.104 | 0.037 | 0.307 |
| *Interaction* | 1.293 | 0.038 | 0.214 | 0.202 | 0.007 | 0.965 |
