# Supplementary material for: Contrasting microbial assembly patterns in the woody endosphere of hybrid and non-hybrid Populus trees
Source: PeerJ. 2025 Oct 10;13:e20073. doi: 10.7717/peerj.20073 (PMC12517286; doi:10.7717/peerj.20073)
Supplement: Supplemental Information 13 — The community matrices were Hellinger transformed prior to computing Bray–Curtis and weighted UniFrac distances. Bolded values highlight significant factors (p < 0.05). [file peerj-13-20073-s013.docx]

|  | **Bray-Curtis distance** | | | **Weighted UniFrac distance** | | |
| --- | --- | --- | --- | --- | --- | --- |
| **Model** | **Sum Sq** | **F-value** | **p-value** | **Sum Sq** | **F-value** | **p-value** |
| **Fungal community** |  |  |  |  |  |  |
| *Host identity* | 0.053 | 3.339 | 0.081 | 0.020 | 2.124 | 0.166 |
| *Sites* | 0.006 | 0.465 | 0.499 | 0.004 | 0.435 | 0.520 |
| *Interaction* | 0.095 | 1.598 | 0.227 | 0.039 | 1.158 | 0.356 |
