# Supplementary material for: Contrasting microbial assembly patterns in the woody endosphere of hybrid and non-hybrid Populus trees
Source: PeerJ. 2025 Oct 10;13:e20073. doi: 10.7717/peerj.20073 (PMC12517286; doi:10.7717/peerj.20073)
Supplement: Supplemental Information 1 — Data was generated using the FastTree 2 algorithm in QIIME2 with a midpoint root. [file peerj-13-20073-s001.pdf]

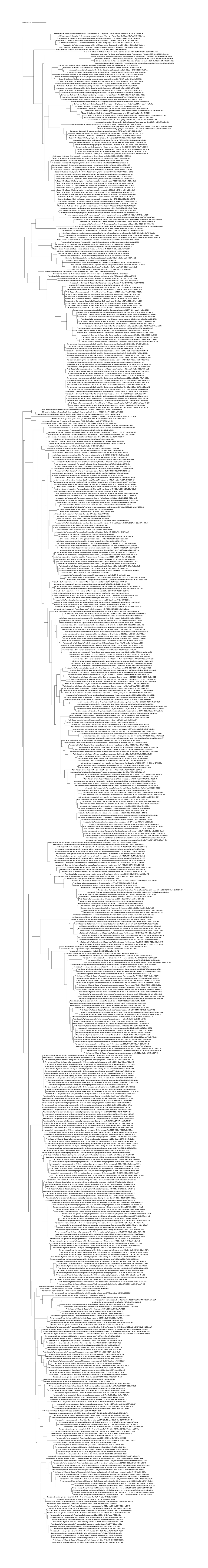
Tree scale: 0.1
Acidobacteriota Acidobacteriae Acidobacteriales Acidobacteriaceae -Subgroup 1- Granulicella x73ebdd1f9f0269b5f8fe94594d1b0acf
Acidobacteriota Acidobacteriae Acidobacteriales Acidobacteriaceae -Subgroup 1- Terriglobus x210bbebbc95d7fdc64f805300c6faa3c
Acidobacteriota Acidobacteriae Acidobacteriales Acidobacteriaceae -Subgroup 1- x05e31c7e1c7a74f33be494583e50ef3c
Acidobacteriota Acidobacteriae Acidobacteriales Acidobacteriaceae -Subgroup 1- x4450657e106cacb78d7093a14357a9c4
Acidobacteriota Acidobacteriae Acidobacteriales Acidobacteriaceae -Subgroup 1- xb42279a0d4b06560b11f32e5b4b31b76
Acidobacteriota Acidobacteriae Acidobacteriales Acidobacteriaceae -Subgroup 1- x36c5099c01ca440d282e2b3979ade46d
Acidobacteriota Acidobacteriae Acidobacteriales Acidobacteriaceae -Subgroup 1- x074aaecdd745814e870d0af71e1ab097
Actinobacteriota Actinobacteria Frankiales Geodermatophilaceae xa7b0674fe9b2832ab10365ab848b2e92
Bacteroidota Bacteroidia Bacteroidales Prevotellaceae Alloprevotella xf902d56403427ed9649b98b291c431e2
Bacteroidota Bacteroidia Flavobacteriales Flavobacteriaceae Flavobacterium x71db38e496ff2f123002350b46ee2a24
Bacteroidota Bacteroidia Flavobacteriales Weeksellaceae Chryseobacterium x1fef3300c568ef6994ea9f868a11be78
Bacteroidota Bacteroidia Flavobacteriales Weeksellaceae Chryseobacterium xfc61e08eda26041fa82012dfa8a1cbdd
Bacteroidota Bacteroidia Flavobacteriales Weeksellaceae Chryseobacterium xd9c0b6fac80c502c24e399b8321d73c5
Bacteroidota Bacteroidia Flavobacteriales Weeksellaceae Chryseobacterium xcaedb66337bacbf2a94b5b58229f389c
Bacteroidota Bacteroidia Sphingobacteriales Sphingobacteriaceae Pedobacter x47ccb9a2a9168dac821b21ee197ffd50
Bacteroidota Bacteroidia Sphingobacteriales Sphingobacteriaceae Pedobacter x9f9c0f61aae89593977fd02d30726cb9
Bacteroidota Bacteroidia Sphingobacteriales Sphingobacteriaceae Pedobacter xe2b82485ea8645463a95806b97b17a6e
Bacteroidota Bacteroidia Sphingobacteriales Sphingobacteriaceae Pedobacter xd4b3eb1b49c61d8c017cde7750b84809
Bacteroidota Bacteroidia Sphingobacteriales Sphingobacteriaceae Sphingobacterium xac0cc0ddb8622633a834c07caba55861
Bacteroidota Bacteroidia Sphingobacteriales Sphingobacteriaceae Mucilaginibacter x5d4c5a9cb71e4a33bc6693f345ac8098
Bacteroidota Bacteroidia Sphingobacteriales Sphingobacteriaceae Mucilaginibacter x0904784f8f54dd2e9c94a17ba94f7780
Bacteroidota Bacteroidia Sphingobacteriales Sphingobacteriaceae Mucilaginibacter x5f8d070a1ab12e82822d856085c98f5d
Bacteroidota Bacteroidia Sphingobacteriales Sphingobacteriaceae Mucilaginibacter xdcbb8c5ee25a1ff94fc27bdb30bb2dc6
Bacteroidota Bacteroidia Sphingobacteriales Sphingobacteriaceae Mucilaginibacter xc91f73e87f086f4f1f80a001c592cd47
Bacteroidota Bacteroidia Sphingobacteriales Sphingobacteriaceae Mucilaginibacter xabf559e1d94c2c0ee173d39a3738e881
Bacteroidota Bacteroidia Sphingobacteriales Sphingobacteriaceae Mucilaginibacter x430e1c7753fbbf3fe669038a981601f6
Bacteroidota Bacteroidia Sphingobacteriales Sphingobacteriaceae Mucilaginibacter xc0fe0c849d416fd5b52ac19ebfda0cdd
Bacteroidota Bacteroidia Sphingobacteriales Sphingobacteriaceae Mucilaginibacter xda3d02902123a7bb49e954195ae1339e
Bacteroidota Bacteroidia Sphingobacteriales Sphingobacteriaceae Mucilaginibacter xbb8dbffb9d97534d02353afe840d8e11
Bacteroidota Bacteroidia Sphingobacteriales Sphingobacteriaceae Mucilaginibacter xceff4f444788df008c2df801df7798ec
Bacteroidota Bacteroidia Chitinophagales Chitinophagaceae Edaphobaculum x56d9bff0dcfc19388ba6808ddfacf03e
Bacteroidota Bacteroidia Chitinophagales Chitinophagaceae Edaphobaculum xe8bc6e681aef294ce54eba5de4d91cba
Bacteroidota Bacteroidia Chitinophagales Chitinophagaceae xa4bc17a9e95e9f0f7668594a5e5888cd
Bacteroidota Bacteroidia Chitinophagales Chitinophagaceae x8a8e871b49367a6ac1adb770994ac3b6
Bacteroidota Bacteroidia Chitinophagales Chitinophagaceae Aurantisolimonas xb32ae3858bfe509e46efda81f6b6daae
Bacteroidota Bacteroidia Chitinophagales Chitinophagaceae x64e48666eac3d9da6106efad47fd7804
Bacteroidota Bacteroidia Chitinophagales Chitinophagaceae Chitinophaga xb061894337ae10139e8494709a65d256
Bacteroidota Bacteroidia Chitinophagales Chitinophagaceae x918b4a5d8d6db4b1d722da3d7d851584
Bacteroidota Bacteroidia Chitinophagales Chitinophagaceae x46595385b2339dab660636e60d851491
Bacteroidota Bacteroidia Chitinophagales Chitinophagaceae Terrimonas xea30f2d967f9fdc4a65137f2cd6b6af2
