# Supplementary material for: Contrasting microbial assembly patterns in the woody endosphere of hybrid and non-hybrid Populus trees
Source: PeerJ. 2025 Oct 10;13:e20073. doi: 10.7717/peerj.20073 (PMC12517286; doi:10.7717/peerj.20073)
Supplement: Supplemental Information 2 — Data was generated using the FastTree 2 algorithm in QIIME2 with a midpoint root. [file peerj-13-20073-s002.pdf]

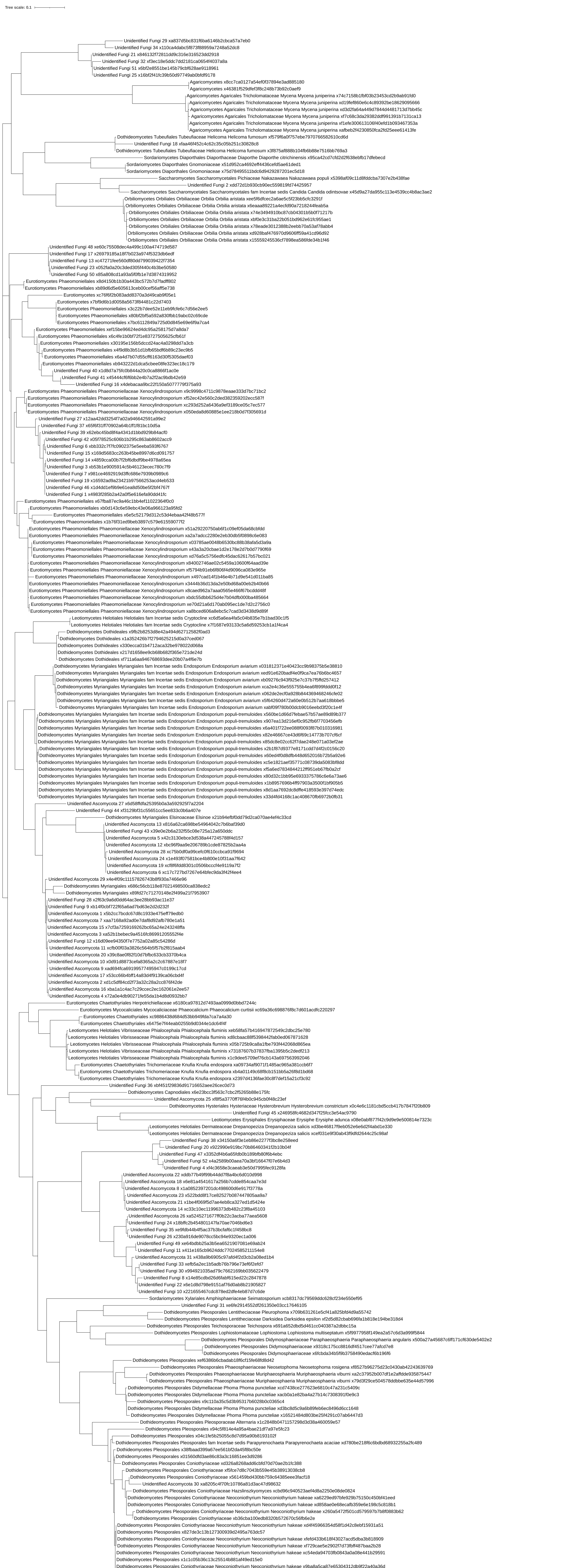
Tree scale: 0.1
